# Peuzt – Jeghers syndrome with gastric type mucinous endocervical adenocarcinoma in a young woman: A case report

**DOI:** 10.1016/j.amsu.2021.102700

**Published:** 2021-08-10

**Authors:** Giap Vu Dinh, Nhat Doan Thi Hong, Tu Vo Ngoc, Long Nguyen Thanh, Hoai Hoang Thi, Huyen Phung Thi

**Affiliations:** a Department of Breast and Gynecologic Surgical Oncology, Nghe An Oncology Hospital, Nghe An, Viet Nam; b Deapartment of Clinical Surgery, Vinh Medical University, Nghe An, Viet Nam; c Department of Oncology, Hanoi Medical University, Hanoi, Viet Nam; d Department of Medical Oncology 6, Vietnam National Cancer Hospital, Hanoi, Viet Nam

**Keywords:** Adenocarcinoma, Peutz-Jeghers syndrome, Gastric type mucinous endocervical adenocarcinoma, Case report

## Abstract

**Introduction:**

Patients with Peutz-Jeghers syndrome (PJS) have high risk of malignancies, including gynecological cancers. Gastric type mucinous cervical adenocarcinoma might be presented in about 11–17% of PJS patients but the literature about this is limited.

**Case presentation:**

We presented a rare case of a 39-year-old Vietnamese woman with Peutz-Jeghers syndrome who has gastric type adenocarcinoma (GAS) of the cervix. She underwent radical hysterectomy, and the diagnosis was confirmed on final pathology.

**Conclusions:**

Oncologists and pathologists should recognize this rare clinical scenario for early diagnosis and treatment. This subtype also has an aggressive nature and poor prognosis.

## Highlights

• Gastric type adenocarcinoma of the uterine cervix (GAS) is a rare variant of endocervical adenocarcinoma.
• An association exists between GAS and Peutz-Jeghers syndrome.
• Careful evaluation by gynecological oncologists is needed for early detection and treatment of this condition.

## 1 Introduction

Although cervical squamous cell carcinoma (SCC) is generally more common than adenocarcinoma, the incidence of SCC significantly decreased due to cervical cancer screening programs [1]. Gastric type mucinous adenocarcinoma (GAS) is the most frequent subtype of adenocarcinoma which is unrelated to HPV infection, accounting for approximately 10% of endocervical carcinoma in the general population and up to 25% in Japanese population [2]. GAS is classified as a new variant of endocervical adenocarcinoma (ECA) with distinct morphologic and phenotypical features, metastatic patterns and has a worse prognosis compared to the usual HPV-associated type cervical adenocarcinoma [3].

Peutz-Jeghers syndrome (PJS) is a rare autosomal dominant disorder with an incidence between 1/200.000 and 1/50.000live births [4,5]. PJS is associated with the growth of gastrointestinal hamartomatous polyps and pigmentation around mouth, nose, anus, oral mucosa and fingers. The genetic landmark of PJS is STK-11/LKB1 mutation, allowing early detection and screening of family members. Gastric type mucinous cervical adenocarcinoma might be presented in about 11–17% of PJS patients [6,7]. We reported a rare and interesting case of GAS in a 39 year-old Vietnamese woman with Peutz-Jeghers syndrome. This work has been reported in line with the SCARE 2020 criteria [8].

## 2 Case presentation

A 39-year-old woman visited at a provincial oncology center with a complaint of vaginal discharge and chronic pelvic pain for 4 months. The patient got married and was nulliparous. She had a history of intestinal multiple polyp resection by endoscopy 7 years ago, with no history of smoking, drug or alcohol use. Her father and her brother had been diagnosed with Peutz-Jeghers syndrome and died of colon cancer at the age of 64 and 45, respectively. Her older brother and his two children had experienced laparoscopic surgery for strangulated intussusception of the colon. She meets the diagnostic criteria for Peutz-Jeghers syndrome proposed by WHO criteria for diagnosis with gastrointestinal hamartomatous polyps (Fig. 2), characteristic, prominent, mucocutaneous pigmentation (Fig. 3) and family history of Peutz-Jeghers syndrome. The patient did not have screening PAP smear before.

**Fig. 1:**
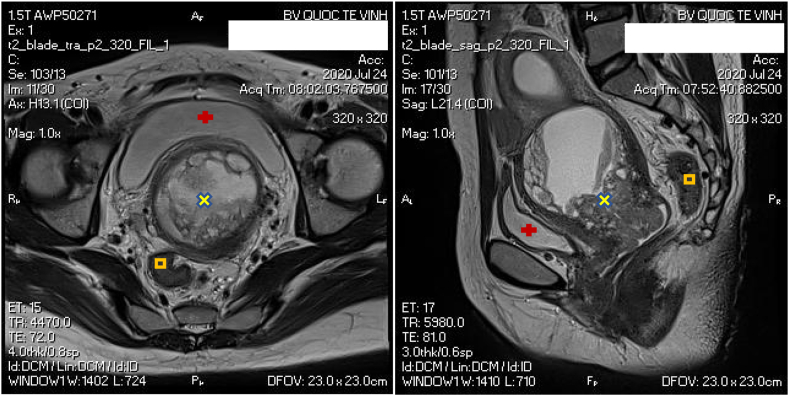
Pelvic magnetic resonance image showing an enlargement of the cervix with pelvic lymph nodes of about 8 × 9mm in size and a slightly enlarged uterus with a fluid filled uterine corpus (about 51–70mm). The cervical parametrium is intact. (x: the tumor, +: bladder, □: rectum).

**Fig. 2:**
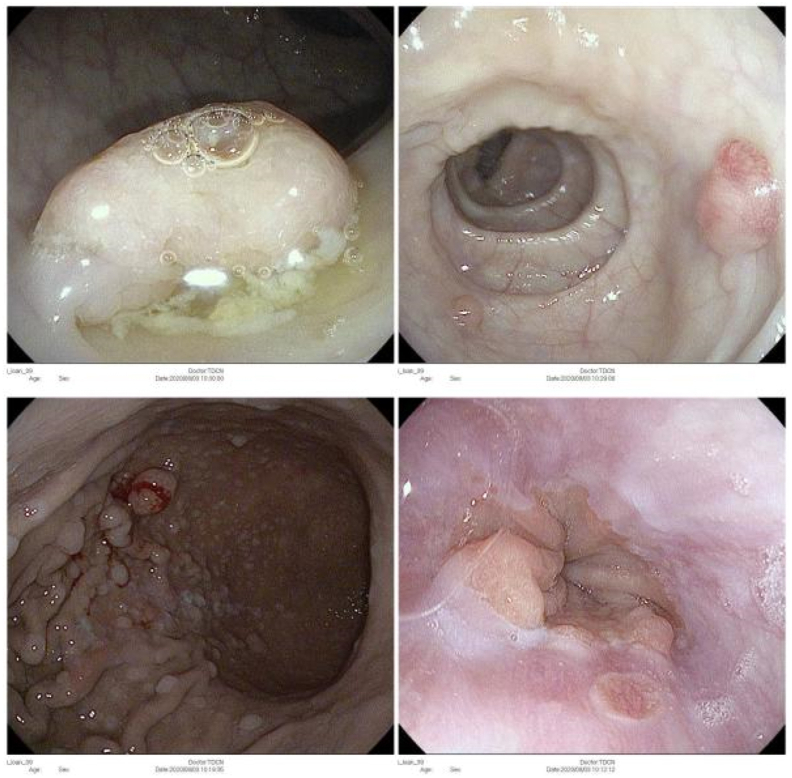
Endoscopy image with hamartomatous polyps of the gastrointestinal tract.

**Fig. 3:**
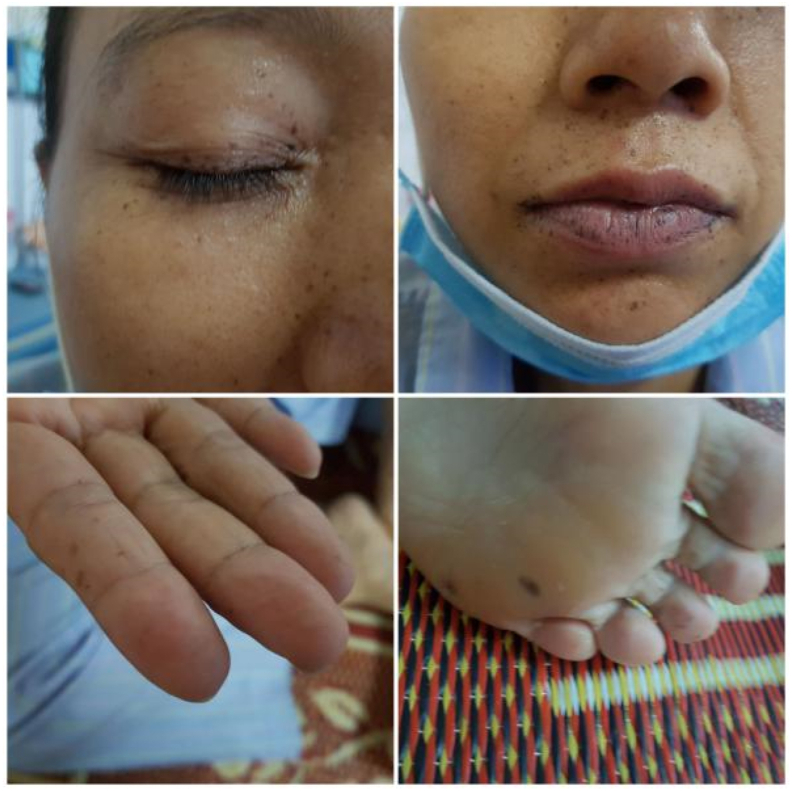
Signs of PJS appear with the development of pigmented areas on the skin and in the mouth, called mucocutaneous hyperpigmentation, dark brown freckling around the mouth and on the lips, eyes, fingers, toes. (For interpretation of the references to colour in this figure legend, the reader is referred to the Web version of this article.)

Gynecological examination revealed bloody vaginal discharge, a mass 3 × 4 cm in diameter was found on the cervix, not invading vagina and parametrium and no evidence of metastatic lymphadenopathy involvement. Initial biopsy was negative and colposcopy-guided cervical biopsy of the red ulcerated lesion was performed, and atypical glandular cells (AGC), endocervical type was recorded on histopathological examination.

Sonographic findings included a slightly enlarged uterus with a fluid filled uterine corpus (about 51–70mm). Endometrial biopsy was performed, which yielded endometrial hyperplasia. Pelvic magnetic resonance imaging (MRI) revealed a prominent uterine cervix with a diameter of 4 × 2.3 × 3.5cm (Fig. 1). Some pelvic lymph nodes about 8 × 9mm in size were found. The mass was confined to the cervix and did not invade the parametrium. No evidence of distant metastasis was identified. Laboratory data showed no blood, urine changes and serum tumor markers were within their normal ranges.

**Fig. 4:**
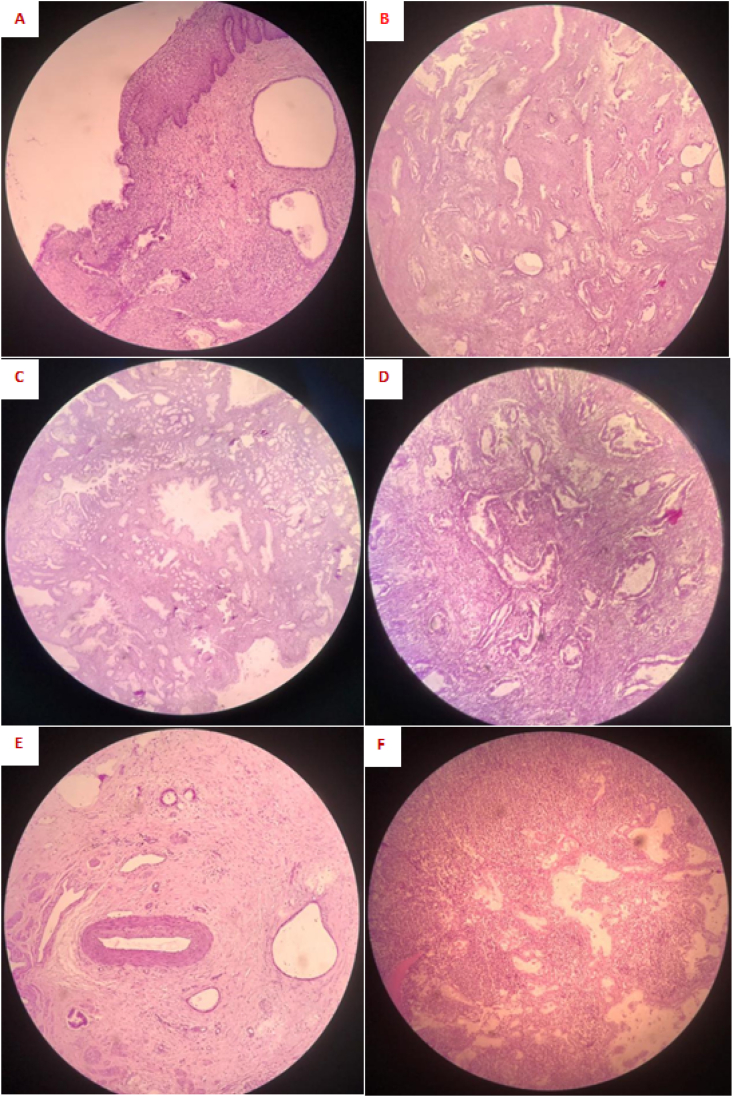
Histologic images: Malignant lesion comes from junctional zone (A). Low power view of gastric type adenocarcinoma composed of variable shaped and size glands (B, C), lined by columnar cells with abundant, mucin-producing cytoplasm, and irregular nuclei (D). Composed of malignant gland-like structures is seen in stromal (E) and lymph node (F).

The patient was then diagnosed with clinical stage IB3 cervical cancer in accordance with the International Federation of Gynecology and Obstetrics 2018 staging system. Specific findings were 1) a tumor size of 4 cm in the greatest dimension, 2) depth of invasion of 3.5 cm, 3) no lymphovascular invasion, 4) no involvement of parametrium and 5) extension to the endometrium. Eventually, the patient underwent radical transabdominal hysterectomy, bilateral salpingo-oophorectomy and bilateral pelvic lymphadenectomy by a senior surgeon specialized in gynecological cancer surgery without delay. The final histopathological analysis of the specimen from radical surgery confirmed gastric type mucinous adenocarcinoma of the cervix (Fig. 4). Pathological stage IIIC was diagnosed with tumor dimension 5cm, myometrial invasion and metastasis to pelvic lymph nodes (2/7 positive nodes). The patient then had adjuvant chemoradiation therapy. After one year of follow up, the patient was stable and no recurrences were detected.

## 3 Disscussion

PJS patient have a significantly increased overall lifetime risk of gastrointestinal and extra-gastrointestinal cancers including, cancer of stomach (29%); colon (39%); breast (54%), lung (15%), uterus (9%), ovary (21%) [5,9,10]. Therefore, if patients with Peutz-Jeghers syndrome complain of atypical gynecological symptoms such as abdominal pain, watery vaginal discharge and abnormal vaginal bleeding, careful gynecological examination and close monitoring should be performed to ensure early detection of cervical cancer. Besides, GAS is a non-HPV related cervical tumor of which Papanicolaou test has low sensitivity [11]. Therefore, the diagnosis approach of GAS differs from other HPV-associated cervical cancer and MRI, ultrasound or endocervical curettage might be helpful [11].

On the other hand, although clinical symptoms of Peutz-Jeghers syndrome (PJS) such as mucocutaneous pigmentation or multiple polyps have high diagnostic values, the diagnosis of PJS might be missed or delayed due to the rarity of the syndrome [4,5]. Srivatsa et al. reported a case in which pigmentation and PJS were only noticed at the time cervical cancer had already metastasized [12]. Therefore, clinicians should be aware of this association for early diagnosis and treatment.

Minimal deviation adenocarcinoma (MDA), which is an extremely well-differentiated variant of GAS, was first described by Gusserow in 1870 [13]. In 2014, gastric type adenocarcinoma (GAS) was recognized by WHO as a new pathological variant of mucinous adenocarcinoma with distinctive histologic morphology and immunohistochemical (IHC) profile. Pathology specimen of GAS is dominated by cells showing eosinophilic and voluminous cytoplasm with distinct cell borders [14]. In difficult cases, IHC could aid the diagnosis, in which the tumor is positive with MUC6 and HIK1083, and negative with p16 [15]. Compared to usual HPV associated cervical adenocarcinoma, GAS has a more aggressive clinical behavior and a significantly worse prognosis. In a retrospective review of Karamurzin et al., the 5-year and 10-year disease-specific survival of patients with GAS were 42% and 31%, respectively, compared to 91% and 81% in those with usual HPV associated endocervical adenocarcinoma [3].

This patient has pelvic lymph nodes of 8 × 9 mm in diameter, which did not meet the commonly used criteria of lymph node metastasis in cervical cancer (short axis ≥10mm) [16] and PET/CT is unavailable in our hospital to confirm the pelvic lymph nodes were metastatic or not. The tumor size was also borderline (4 cm). Because brachytherapy has not been available in our institution yet, surgery was preferred in these borderline cases. Besides, due to the higher risk of ovarian metastases with cervical adenocarcinoma compared with squamous cell carcinoma (5.0 versus 0.8% in one series), bilateral oophorectomy was also performed [17]. Ovarian preservation or egg freezing might be considered in patients with pregnancy desire after a thorough discussion.

This is among the few cases of cervical gastric type adenocarcinoma associated with Peutz-Jeghers syndrome reported in the literature. Oncologists and pathologists should recognize this rare clinical scenario for early diagnosis and treatment. This subtype also has an aggressive nature and poor prognosis.

## Consent

Written informed consent was obtained from the patient for publication of this case report and accompanying images. A copy of the written consent is available for review by the Editor-in-Chief of this journal on request.

## Funding

The authors received no financial support for the research, authorship, and/or publication of this report.

## Guarantor

Doan Thi Hong Nhat, MD, MSc.

## Ethical approval

The study was approved by Ethics Committee of Vinh Medical University.

## Author contributions

Doan Thi Hong Nhat: main doctor who treated the patient, wrote manuscript. Vu Dinh Giap: took part in the operation, wrote manuscript. Vo Ngoc Tu: took part in the operation, wrote manuscript. Nguyen Thanh Long: followed up the patient, wrote manuscript. Hoang Thi Hoai: took part in the operation, wrote manuscript Phung Thi Huyen: provide consultation, wrote and revise manuscript.

## Trial registry number

This is not a first-in-human study, thus it is not needed.

## Provenance and peer review

Not commissioned, externally peer-reviewed.

## Declaration of competing interest

The authors declare no potential conflicts of interest with respect to the research, authorship, and/or publication of this paper. All authors read and approved the final manuscript for publication.
